# A Novel PET Imaging Using ^**64**^Cu-Labeled Monoclonal Antibody against Mesothelin Commonly Expressed on Cancer Cells

**DOI:** 10.1155/2015/268172

**Published:** 2015-03-25

**Authors:** Kazuko Kobayashi, Takanori Sasaki, Fumiaki Takenaka, Hiromasa Yakushiji, Yoshihiro Fujii, Yoshiro Kishi, Shoichi Kita, Lianhua Shen, Hiromi Kumon, Eiji Matsuura

**Affiliations:** ^1^Department of Cell Chemistry, Okayama University Graduate School of Medicine, Dentistry, and Pharmaceutical Sciences, Okayama 700-8558, Japan; ^2^Department of Urology, Okayama University Graduate School of Medicine, Dentistry, and Pharmaceutical Sciences, Okayama 700-8558, Japan; ^3^Collaborative Research Center for OMIC, Okayama University Graduate School of Medicine, Dentistry, and Pharmaceutical Sciences, Okayama 700-8558, Japan; ^4^Department of Research and Development, Ina Institute, Medical & Biological Laboratories, Co., Ltd., Ina 396-0002, Japan; ^5^Technology Research Laboratory, Shimadzu Corporation, Kyoto 604-8511, Japan

## Abstract

Mesothelin (MSLN) is a 40-kDa cell differentiation-associated glycoprotein appearing with carcinogenesis and is highly expressed in many human cancers, including the majority of pancreatic adenocarcinomas, ovarian cancers, and mesotheliomas, while its expression in normal tissue is limited to mesothelial cells lining the pleura, pericardium, and peritoneum. Clone 11-25 is a murine hybridoma secreting monoclonal antibody (mAb) against human MSLN. In this study, we applied the 11-25 mAb to *in vivo* imaging to detect MSLN-expressing tumors. In *in vitro* and *ex vivo* immunochemical studies, we demonstrated specificity of 11-25 mAb to membranous MSLN expressed on several pancreatic cancer cells. We showed the accumulation of Alexa Fluor 750-labeled 11-25 mAb in MSLN-expressing tumor xenografts in athymic nude mice. Then, 11-25 mAb was labeled with ^64^Cu via a chelating agent DOTA and was used in both *in vitro* cell binding assay and *in vivo* positron emission tomography (PET) imaging in the tumor-bearing mice. We confirmed that ^64^Cu-labeled 11-25 mAb highly accumulated in MSLN-expressing tumors as compared to MSLN-negative ones. The ^64^Cu-labeled 11-25 mAb is potentially useful as a PET probe capable of being used for wide range of tumors, rather than ^18^F-FDG that occasionally provides nonspecific accumulation into the inflammatory lesions.

## 1. Introduction

Mesothelin (MSLN) is a 40-kDa cell differentiation-associated glycoprotein appearing with carcinogenesis. MSLN was found as an antigen recognized by the monoclonal antibody (mAb), K1, generated by immunization of mice with the human ovarian carcinoma cell line, OVCAR-3. The protein has been named as MSLN because the expression of MSLN in normal tissue was limited to mesothelial cells lining the pleura, pericardium, and peritoneum [1]. On the contrary, MSLN is widely expressed in human cancers, for example, the majority of ovarian cancers and pancreatic adenocarcinomas, and in 100% of epithelial mesotheliomas. Recent studies showed that it is also found in lung adenocarcinomas, gastric cancers, triple-negative breast cancers, uterine serous carcinoma, acute myeloid leukemia, and cholangiocarcinoma [2–13]. Because of its limited distribution in normal tissues and elevated expression in cancers, MSLN has the potential to become a suitable target for a wide range of cancer diagnosis and therapy by using its specific antibodies.

A precursor of MSLN is encoded as a 622-amino acid glycoprotein and cleaved by furin into a membrane-attached 40-kDa form (MSLN) and a 31-kDa-shed protein, megakaryocyte potentiating factor (MPF). MSLN is attached to cell surface through glycosylphosphatidylinositol linked to its carboxyl terminus [10].

The physiological function of MSLN is not fully elucidated as MSLN-deficient mice are fertile and do not exhibit any apparent phenotype [14]. However, recent studies indicate that MSLN may play an important role in cell adherence, cell survival/proliferation, tumor progression, and chemoresistance [15]. MSLN may aid in the peritoneal implantation and metastasis of tumors through its interaction with CA125 (also known as MUC16), an ovarian cancer antigen [16–18]. MSLN overexpression promotes cancer cell invasion by inducing matrix metalloproteases 7 and 9 [19, 20]. MSLN may also promote cancer cell survival and proliferation via the NF-*κ*B signaling pathway [21]. Overexpression of MSLN constitutively activates NF-*κ*B and it leads to higher interleukin-6 production and induces tumorigenesis [22]. Further, the MSLN expression promotes resistance to certain chemotherapy drugs, such as TNF-*α*, paclitaxel, and a combination of platinum and cyclophosphamide [23, 24].

Since MSLN is overexpressed in a variety of malignancies, it is a good target for anti-MSLN antibody-based diagnosis and therapy. A number of anti-MSLN mAbs have been developed, and MORAb-009 (Amatuximab) is a chimeric (mouse/human) antibody [17]. HN1 [25] was isolated from a human scFv phage display library and converted into a fully intact human IgG.

As trace amount of MSLN can be detected in the blood of some patients with MSLN-positive cancers,* in vitro* diagnostic tests have been developed not only for diagnosis but also for following the course of some of these patients. A murine mAb against MSLN, clone 11-25, was established by immunizing mice with recombinant human MSLN [26]. The 11-25 mAb was utilized in a sandwich ELISA for detecting soluble form of MSLN in sera of patients with mesothelioma. The 11-25 mAb binds to MSLN in soluble form(s) and to a membrane-attached form. Because the soluble form(s) of MSLN is present in very small amount (1.4–3.8 nmol/L) [26], it should not interfere with antibody-based therapies that target the MSLN antigen on cancer cells [2].

Positron emission tomography (PET) is a noninvasive, highly sensitive, and a quantitative tomographic imaging modality. It is clinically important as an imaging tool in cancer diagnosis and staging for a number of malignancies. The antibody-based PET technology is an attractive method for noninvasive tumor detection since this strategy combines the high sensitivity of PET with the high antigen specificity of mAbs [27]. ^64^Cu (*t*_1/2_ = 12.7 h) is the widely used isotope for antibody-based PET, partly due to its wide availability, low cost, and versatile chemistry [28].

As MSLN is overexpressed in a wide range of cancers, anti-MSLN 11-25 mAb has the potential to become a PET imaging agent for detecting various kinds of MSLN-expressing cancers.

In this study, we performed* in vitro* and* in vivo* investigations of anti-MSLN (11-25) mAb to evaluate its utility as an imaging probe for detecting MSLN-expressing tumors. To apply to PET imaging, we labeled DOTA-conjugated 11-25 mAb with positron-emitting ^64^Cu and monitored* in vivo* distribution through PET imaging of human pancreatic cancer xenografts in nude mice.

## 2. Materials and Methods

### 2.1. Reagents

Mono-N-hydroxysuccinimide ester 1, 4, 7, 10-tetraazacyclododecane-1, 4, 7, 10-tetraacetic acid (DOTA-mono-NHS ester) was purchased from the Macrocyclics (Dallas, TX). PD-10 desalting columns were purchased from GE Healthcare (Uppsala, Sweden). Amicon Ultra 0.5 centrifugal filter units were purchased from Merck Millipore (Billerica, MA). All of other chemicals used in the present study were of reagent grade.

### 2.2. Anti-MSLN mAb

Anti-MSLN mAb 11-25 (IgG2b, *κ*) was prepared from the culture media of hybridoma clone 11-25, which was previously generated by immunizing mice with a recombinant human MSLN protein, as described [26]. Briefly, BALB/c mice were immunized with recombinant MSLN (1057–1908 bp region of NM_005823: secretory extracellular domain), mixed with Freund's complete adjuvant for first immunization, and boosted 4 times with the same antigen emulsified with the incomplete adjuvant. Splenocytes from the immunized mice were fused with P3U1 myeloma cells. Hybridoma cells producing specific mAb against MSLN were cloned. The MSLN-specific antibodies were purified on a protein A column from the culture fluid of hybridoma cells. Among the purified mAbs, 11-25 mAb showed the highest reactivity in immunohistochemistry and flow cytometry. As IgG2b isotype-matched control, mouse mAb against keyhole limpet hemocyanin (KLH) was purchased from R&D Systems (Minneapolis, MN).

### 2.3. Cell Culture

Human pancreatic cancer cell lines, BxPC-3, CFPAC-1, and PANC-1, human lung cancer cell lines, NCI-H226, MSTO-211H, and NCI-H520, and a human epidermoid carcinoma, A-431, were purchased from American Type Culture Collection (ATCC, Rockville, MD) and were maintained in RPMI-1640 (for BxPC-3, H226, 211H, and H520), Iscove's modified Dulbecco's medium (IMDM; for CFPAC-1), and Dulbecco's Modified Eagle Medium (DMEM; for PANC-1), containing 10% fetal bovine serum, 1% penicillin/streptomycin, in a humidified incubator maintained at 37°C with 5% CO_2_.

### 2.4. Soluble MSLN Determination

The concentration of the soluble form of MSLN, in conditioned culture media of cancer cell lines, was determined by a sandwich ELISA in a similar way, as previously reported [26]. Ninety-six-well microtiter plates (MaxiSorp, Thermo Fisher Scientific Inc., Waltham, MA) were coated with anti-MSLN mAb, 14–30 (5 *μ*g/mL) at 4°C overnight. The plates were blocked with PBS containing 1.0% BSA for 1 hour. Each cell line was cultured for 5 days in their appropriate culture medium and the conditioned medium was collected by centrifugation. The conditioned medium was diluted five- and tenfold with PBS containing 1% BSA, 0.1% Tween-20, and added to the wells. After washing with PBS containing 0.1% Tween-20, the wells were incubated for 1 hour with biotinylated mAb, 11-25, and subsequently reacted for 30 minutes with avidin-conjugated peroxidase (DAKO, Glostrup, Denmark) diluted to 1 : 20,000. Followed by four washes with PBS, 100 *μ*L/well tetramethylbenzidine (TMB) (Moss Inc., Pasadena, MD) was added and incubated for 30 minutes. The color development was stopped by the addition of 0.36 N H_2_SO_4_. Soluble MSLN concentration was calculated by referring to a standard curve using serially diluted recombinant MSLN.

### 2.5. Western Blot Analysis

Western blot analysis was performed with cancer cells, that is, BxPC-3, CFPAC-1, PANC-1, and A-431. From each cancer cell line, total proteins were extracted with RIPA buffer (25 mM Tris-HCl pH 7.6, 1% NP-40, 0.1% DOC, 0.1% SDS, 0.15 M NaCl, 1 mM EDTA, 10 mg/L leupeptin, and 1 mM PMSF) and electrophoresed on 4–15% SDS-polyacrylamide gradient gel (Bio-Rad). Proteins were subsequently transferred to nitrocellulose membrane (Life Technologies). After blocking with 5% skimmed milk in 20 mM Tris-HCl pH 7.5, 500 mM NaCl, 0.05% (v/v) Tween-20 (TBS-T) for 1 hour, the blot membrane was incubated with 10 *μ*g/mL of 11-25 mAb or mouse anti-*β* action mAb, AC-15 (Sigma), at 4°C for 18 hours. The membrane was then incubated with peroxidase-labeled anti-mouse IgG F(ab′)_2_ (Rockland, Gilbertsville, PA) for 12 hours at 4°C. After washing with TBS-T buffer, the color was developed with DAB. The area of each band was measured with the ImageJ software (National Institutes of Health, Bethesda, MD).

### 2.6. Semiquantitative Reverse Transcription PCR

Semiquantitative reverse transcription PCR was performed to analyze the expression of MSLN in BxPC-3, CFPAC-1, PANC-1, and A-431 cells. Total RNA was extracted from cancer cell lines using Trizol reagent (Life Technologies Corporation, Carlsbad, CA) after standard protocol. MSLN- and glyceraldehyde-3-phosphate dehydrogenase- (GAPDH-) mRNA were first reverse-transcribed to cDNA. Semiquantitative PCR was carried out using the LightCycler real-time detection system (Roche Diagnostics, Mannheim, Germany), according to the manufacturer's instructions, for 50 cycles of 10 seconds (s) at 95°C, 2 s at 68°C, and 10 s at 72°C. 5′-CTATTCCTCAACCCAGATGCGT and 5′-GCACATCAGCCTCGCTCA were the primers used to detect mesothelin. GAPDH was used as an internal control.

### 2.7. Flow Cytometric Analysis for Expression of Mesothelin

Cultures of BxPC-3, CFPAC-1, PANC-1, and A-431 cells were harvested into single-cell suspension by treatment with PBS containing 0.1% collagenase type IV (Invitrogen) and 1 mM EDTA. The 1 × 10^6^ cells were washed once with cold PBS and incubated for 1 hour on ice in the presence of the 11-25 mAb at 5 *μ*g/mL in PBS containing 2% FBS and 1 mM EDTA. As control, cells were treated with isotype control (IgG2b) at 4 *μ*g/mL. Then, the cells were washed twice and incubated with goat anti-mouse IgG labeled with Alexa Fluor 488 for 1 hour on ice. The cells were washed twice and suspended in 0.5 mL PBS containing 2 *μ*g/mL propidium iodide and 1 mM EDTA before analysis with a FACSAria flow cytometer (BD Biosciences).

### 2.8. Immunofluorescence Staining and Confocal Microscopy

BxPC-3, CFPAC-1, and PANC-1 cells were plated onto 8-well chamber slides at a concentration of 2 × 10^4^ cells/0.2 mL/well and incubated for 3 days at 37°C with 5% CO_2_. The cells were washed three times with serum-free medium and fixed with 4% formaldehyde in PBS at room temperature (RT) for 10 minutes. The cells were washed with PBS containing 10 mM glycine and blocked with Dako Protein Block for 10 minutes. Then, the cells were treated with 5 *μ*g/mL anti-MSLN mAb overnight at 4°C. The cells were washed with PBS and incubated with FITC-labeled anti-mouse IgG (Life Technologies), Alexa Fluor 594-labeled wheat germ agglutinin (WGA) (Life Technologies), and 4′, 6-diamidino-2-phenylindole dihydrochloride (DAPI) (Dojindo, Kumamoto, Japan) for 30 minutes. Cells were imaged using a confocal laser scanning microscope, LSM510 (ZEISS, Oberkochen, Germany). For staining of cancer xenografts, the cancer cells were inoculated to nude mice, as described in the Animal Model Section, and the resulting tumors were taken from the mice, soaked in OCT compound, and frozen. The frozen sections were prepared with cryostat and stained with Alexa Fluor 488-labeled 11-25 mAb, Alexa Fluor 594-labeled WGA, and DAPI.

### 2.9. Animal Model

All animal experiments were conducted in accordance with guidelines of Okayama University and approved by University's Animal Care and Use Committee (OKU-2013098). Five-week-old male BALB/c nude mice were purchased from Charles River (Tokyo, Japan) and were maintained under specific pathogen-free conditions at Okayama University before use. For* in vivo* fluorescent imaging, male BALB/c* nu/nu* mice (8-9 weeks old) were inoculated subcutaneously with PANC-1 (1 × 10^7^ cells) in the right thigh and BxPC-3 (5 × 10^6^ cells) in the left thigh. For PET imaging, male BALB/c nu/nu mice (9-10 weeks old) were inoculated with CFPAC-1 (3 × 10^6^ cells) in the right shoulder as well as PANC-1 in the right thigh and BxPC-3 in the left thigh. The imaging experiments were performed when the tumors became about 8 mm in diameter.

### 2.10. Labeling of Alexa Fluor 750 to 11-25 mAb and* In Vivo* Near-Infrared Fluorescence (NIRF) Imaging

The 11-25 mAb was labeled to Alexa Fluor 750 dye, according to the manufacturer's protocols (Molecular Probes Inc., Eugene, OR). Briefly, the antibody (1 mg/mL) in 0.1 M carbonate buffer (pH 8.8) was reacted with Alexa Fluor 750 for 1 hour in the dark. Unlabeled dye was removed by gel filtration on a Sephadex G-25 (GE Healthcare, Uppsala, Sweden) column. Purified Alexa Fluor 750-labeled antibody was filtrated through a 0.2 *μ*m syringe filter and stored at 4°C before use. The absorbance at 280 nm and 752 nm was measured and the degree of labeling (DOL) was calculated after the manufacturer's instructions. The obtained DOL of Alexa Fluor 750-labeled 11-25 mAb was 3.2.

Alexa Fluor 750-labeled 11-25 mAb (90 *μ*g/mouse) was administered via tail vein of mice bearing both BxPC-3 and PANC-1 (*n* = 3). Fluorescence from the labeled Alexa Fluor 750 was then monitored by IVIS-200 imaging system (Xenogen, Alameda, CA) with emission at 680 nm and excitation at 780 nm 24, 48, and 72 hours after intravenous (i.v.) injection. After fluorescence imaging, tumors were dissected and* ex vivo* fluorescence image was taken.

### 2.11. DOTA Conjugation and Radiolabeling of mAbs

DOTA conjugation to mAbs was carried out by incubation in PBS pH 7.0 for 3 hours at a ratio of DOTA-NHS: 11-25 mAb, or control anti-KLH mAb (IgG2b, *κ*), being 100 : 1. The DOTA-conjugated mAbs were separated from unconjugated DOTA on PD-10 column. The number of conjugated DOTA molecules per molecule of IgG was calculated with MALDI-TOF MS (4800 Plus MALDI TOF/TOF Analyzer, AB SCIEX, Framingham, MA) by comparison of average molecular mass of untreated and DOTA-conjugated antibodies. ^64^Cu was produced by a cyclotron (HM-12 cyclotron, Sumitomo Heavy Industries, Ltd., Tokyo, Japan) and purified, according to the previously reported method [29]. For radiolabeling, ^64^CuCl_2_ (480–550 MBq) was diluted with 0.1 M sodium acetate buffer (pH 6.5) and added to 0.51 mg of DOTA-conjugated 11-25 mAb or 0.48 mg of DOTA-anti-KLH mAb, respectively. The reaction mixture was incubated for 1 hour at 40°C with constant shaking. Unreacted ^64^Cu was eliminated by ultrafiltration in Amicon Ultra 0.5 centrifugal filter units. Radiochemical purity was determined by a combination of thin-layer chromatography and autoradiography (TLC-ARG) and HPLC (LC-20, Shimadzu Co., Kyoto, Japan). For TLC-ARG, samples were spotted on silica gel plates (silica gel, 60 RP-18 F254S, Millipore) and developed using 50 mM EDTA (pH 8.0)/methanol = 1/2 as the mobile phase. Radioactivity derived from antibody-bound ^64^Cu stays at the origin and unbound ^64^Cu goes to the top of the chromatogram. HPLC was performed on TSKgel Super SW3000 column (4.6 mm × 30 cm, Tosoh Corp., Tokyo, Japan) with 10 mM phosphate 300 mM NaCl (pH 7.0) as a mobile phase at a flow rate of 0.35 mL/min.* In vitro* stability of the radiolabeled mAbs after 24 and 48 hours of incubation in mouse plasma at 37°C was also analyzed. ^64^Cu-DOTA-11-25 mAb or anti-KLH mAb (50 *μ*L) was added to 450 *μ*L of mouse plasma. After 24- and 48-hour incubation, aliquots of the incubated mixture of radiolabeled mAb with the plasma were injected to HPLC with the Super SW3000 column, the eluate was fractionated, and their radioactivity was counted by a *γ*-counter (AccuFLEX *γ*7001, Hitachi Aloka Medical).

### 2.12. ELISA for Antibody Binding

Titers of 11-25 mAb, DOTA-conjugated 11-25 mAb, and ^64^Cu-DOTA-11-25 mAb were tested by ELISA. The wells of a 96-well microtiter plate were coated with 100 *μ*L of the purified MSLN protein at 1 *μ*g/mL and incubated overnight at 4°C. Wells were blocked with PBS containing 1% BSA for 2 hours. Then serially diluted antibody solution was added and incubated for 1 hour. After three washes with 250 *μ*L PBS containing 0.05% Tween-20, the wells were treated with peroxidase-conjugated goat anti-mouse IgG (Life Technologies) for 1 hour. After washes, 100 *μ*L of TMB-US (Moss Inc., Pasadena, MD) was added and the color was developed. Then, the reaction was stopped by adding 100 *μ*L of 2 N H_2_SO_4_. The absorbance at 450 nm was measured with Sunrise-Basic plate reader (Tecan, Grodig, Austria).

### 2.13. Cell Binding Assay of ^64^Cu-DOTA-11-25 mAb

Assay was performed with live cells using a modification of the method of Li et al. [30]. BxPC-3 cells were transferred to 24-well plates at 5 × 10^4^ cells/well/mL and cultured for 4 days in a CO_2_ incubator at 37°C. The cell number became around 4 × 10^5^ cells/well. Five to eight hundred nM of ^64^Cu-DOTA-11-25 mAb was incubated with the cell monolayers in triplicate for 2 hours on ice in a complete growth medium (RPMI-1640 medium containing 10% fetal bovine serum). The cells were washed twice with 1 mL of ice-cold PBS and lysed by incubation at 37°C in 0.3 mL of 0.2 M sodium hydroxide for 30 min. Aliquots of each sample were analyzed for protein by a bicinchoninic acid assay (Pierce). Samples were transferred into sampling tubes and their radioactivity was counted in a *γ*-counter. Dissociation constant in nM was estimated by nonlinear fitting of the specific binding versus the concentration of ^64^Cu-DOTA-11-25 mAb using Prism software (GraphPad Software, Inc., La Jolla, CA).

### 2.14. Small Animal PET Imaging, Tissue Biodistribution, and* In Vivo* Stability

Each mouse bearing BxPC-3, CFPAC-1, and PANC tumor was anesthetized by inhalation of isoflurane and injected with approximately 11 MBq of ^64^Cu-DOTA-11-25 mAb (15 *μ*g mass) (*n* = 3) or ^64^Cu-DOTA-anti-KLH mAb (11 MBq in 14 *μ*g protein) (*n* = 3) via the tail vein and 30-minute (at 0 and 24 hours) or 60-minute (at 48 hours) static PET scans were performed using a small animal PET scanner (Clairvivo PET, Shimadzu, Kyoto, Japan) and the images were reconstructed using the 3D-DRAMA method. After the PET scans, CT data were acquired at 80 kV and 450 *μ*A with slice thickness of 90 *μ*m by using eXplore CT (GE Healthcare Japan, Tokyo, Japan). PET and CT images were converted into DICOM format, fused, and analyzed using the PMOD software version 3.3 (PMOD Technologies Ltd., Zurich, Switzerland). Following the terminal PET scans at 48 hours after injection, all mice were euthanized for biodistribution studies. The tumors and major organs were removed and weighed. Radioactivity was measured with a gamma-counter. In addition, separate groups of mice were injected with 11 MBq of ^64^Cu-DOTA-11-25 mAb or with ^64^Cu-DOTA-anti-KLH mAb and euthanized at 24 or 48 hours after injection for biodistribution studies. The data were expressed as percentage of injected dose per gram of tissues (%ID/g).* In vivo* stability was measured by TLC. When the mice were sacrificed for biodistribution analysis 24 and 48 hours after injection, an aliquot of the blood was taken and centrifuged at 5,000 ×g for 10 min and 1 *μ*L of the plasma was spotted on the TLC. Also an aliquot of liver and kidney was weighed and three volumes (v/w) of PBS were added and homogenized. The homogenate was centrifuged at 5,000 ×g for 10 min and 1 *μ*L of the supernatant was spotted to the TLC plate. The TLC plate was developed and analyzed as described above.

### 2.15. Statistical Analysis

Data are presented as the mean ± SD. Statistical analysis was performed using a nonpaired Student *t*-test for comparison of 2 groups. Statistical significance was established at *P* < 0.05.

## 3. Results

### 3.1. Soluble MSLN Detection in the Culture Media of Cancer Cells

Table 1 shows the result of soluble MSLN determination in the culture media of cancer cells, determined by a sandwich ELISA. Pancreatic adenocarcinoma cells, CFPAC-1 and BxPC-3, and lung mesothelioma cells, MSTO-211H and NCI-H226, were positive for soluble MSLN. On the other hand, pancreatic carcinoma cells, PANC-1, and lung squamous carcinoma cells, NCI-H520, were negative.

### 3.2. MSLN Expression in Human Cancer Cells

**Figure 1:**
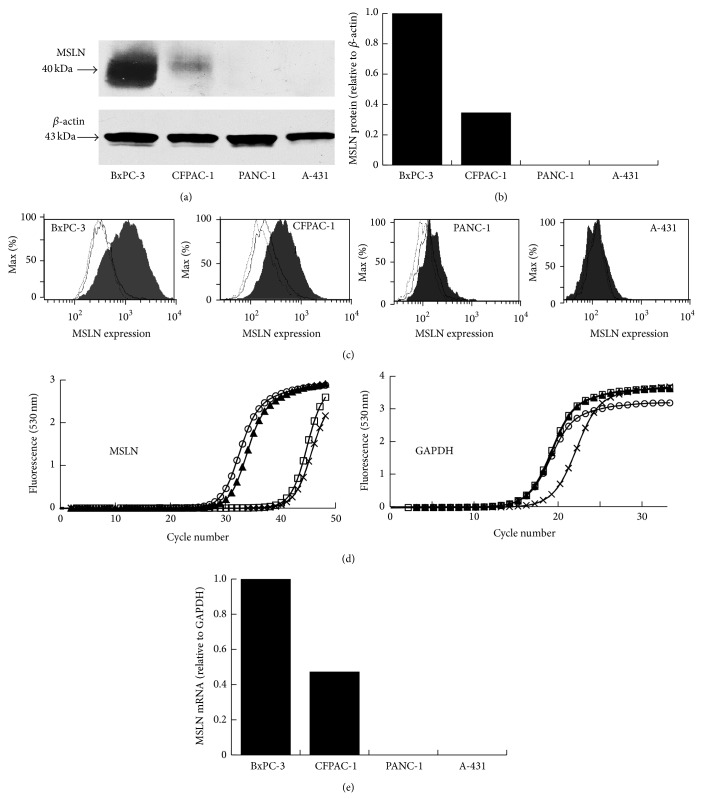
Analysis of MSLN protein expression in cancer cell lines by Western blot (a and b) and by flow cytometry using 11-25 mAb (b). *β*-actin served as a loading control. Expression of MSLN mRNA in cultured BxPC-3 (open circles), CFPAC-1 (closed triangles), PANC-1 (open squares), and A-431 (x-marks) cells (d and e).

To determine the expression of the MSLN protein on the pancreatic carcinoma cells, Western blot analysis was performed.  Figure 1(a) shows that BxPC-3 and CFPAC-1 cells expressed the MSLN protein but PANC-1 and A-431 cells did not. A-431 is human epidermal carcinoma cell line and is known as MSLN-negative [31].  Figure 1(b) shows the expression of MSLN relative to that of *β*-actin.  Figure 1(c) shows the results of flow cytometric analysis of the four cancer cells with anti-MSLN mAb, 11-25. The antibody reacted with 50.9% of BxPC-3 cells, 31.4% of CFPAC-1 cells, 0.6% of PANC-1 cells, and 4.4% of A-431 cells. Next, the expression of MSLN mRNA on the four cell lines was investigated by semi-quantitative PCR (Figures 1(d) and 1(e)). BxPC-3 and CFPAC-1 showed MSLN mRNA expression but PANC-1 and A-431 did not.

**Figure 2:**
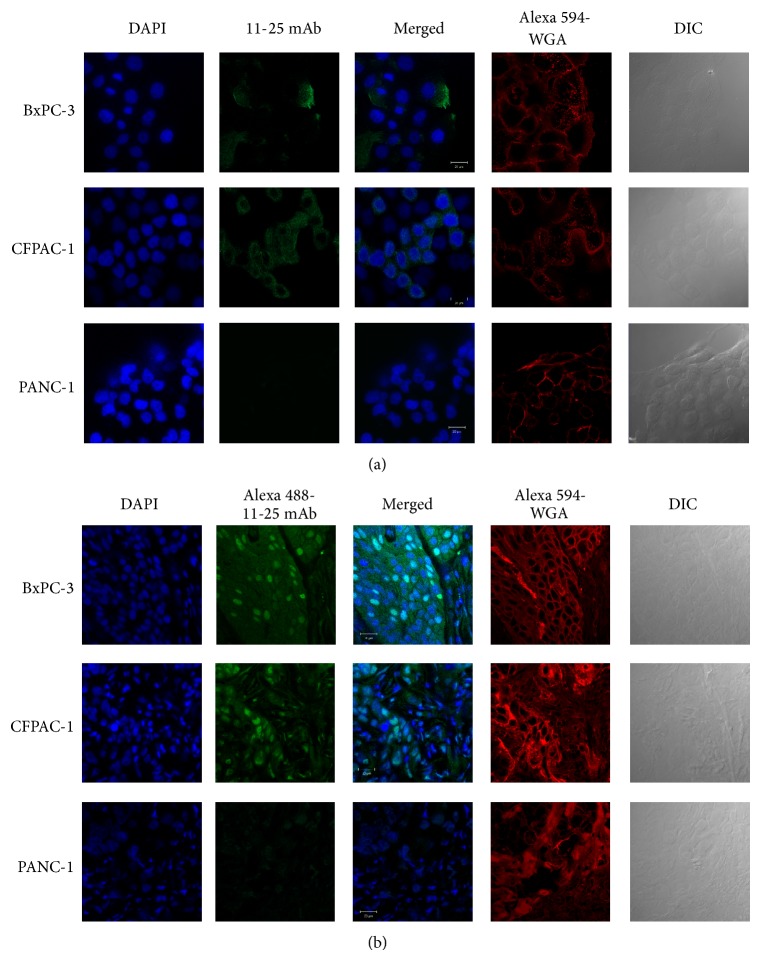
Immunohistochemical analysis of MSLN expression. (a) Expression of MSLN protein in cultured cancer cells detected by immunocytochemistry. BxPC-3, CFPAC-1, and PANC-1 cells were plated in 8-well chamber slides, cultured for 3 days, and incubated with 11-25 mAb followed by treatment with FITC-labeled anti-mouse IgG, Alexa Fluor 594-labeled wheat germ agglutinin (WGA), and DAPI. (b) Expression of MSLN in xenografts. Frozen sections of tumors derived from BxPC-3, CFPAC-1, and PANC-1 were treated with Alexa Fluor 488-labeled 11-25 mAb, Alexa Fluor 594-WGA, and DAPI. Bar: 20 *μ*m.

The binding of 11-25 mAb to the fixed cultured pancreatic carcinoma cells and xenografts in mice was examined by fluorescent immunohistochemical staining. The antibody showed specific binding to both cultured cells and xenografts of CFPAC-1 and BxPC-3 cells, but not to those of PANC-1 cells (Figures 2(a) and 2(b)).

### 3.3. *In Vivo* NIRF Optical Imaging by Alexa Fluor 750-Labeled 11-25 mAb

**Figure 3:**
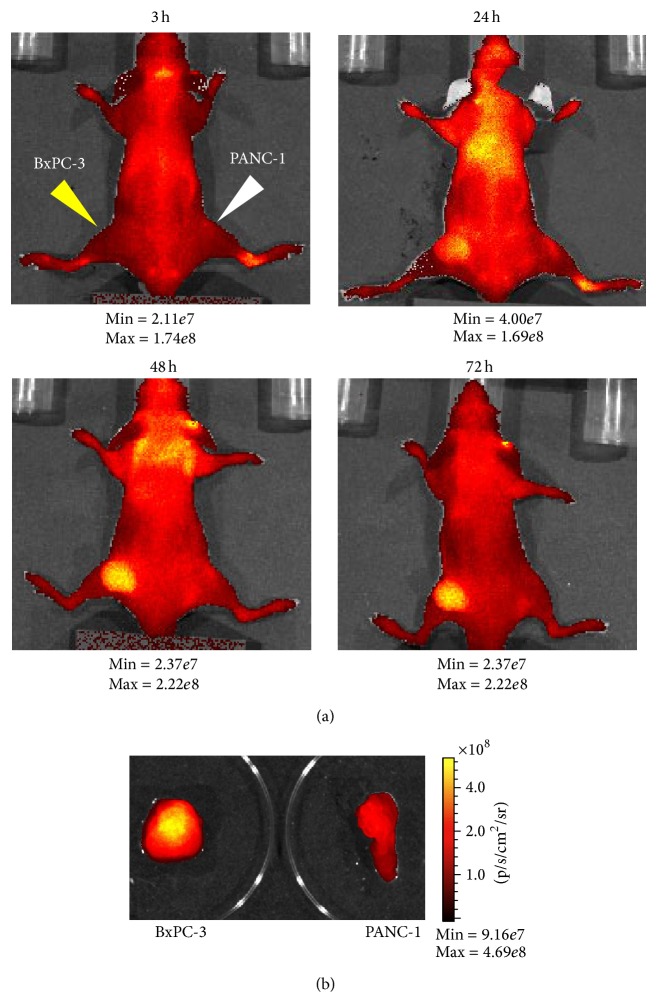
*In vivo* and* ex vivo* NIR optical imaging by Alexa Fluor 750-labeled 11-25 mAb. (a) Alexa Fluor 750-labeled 11-25 mAb (90 *μ*g/mouse) was administered to mice bearing BxPC-3 and PANC-1 intravenously. Alexa Fluor 750 fluorescence was then monitored by IVIS-200 imaging system 24, 48, and 72 hours after the injection. The minimum and maximum values of the photon gage are shown under each photograph. (b) 24 hours after administration of Alexa Fluor 750-labeled 11-25 mAb, tumors were dissected from mice and* ex vivo* fluorescence images were taken.

To analyze the* in vivo* distribution of 11-25 mAb by fluorescence imaging, Alexa Fluor 750-labeled 11-25 mAb was administered to mice bearing BxPC-3 and PANC-1 tumors, and* in vivo* imaging was conducted with IVIS 200.  Figure 3 shows the result of NIRF imaging. At 24 hours after injection, fluorescence from Alexa Fluor 750-labeled 11-25 mAb was detected throughout the body of the animal; however, MSLN-positive BxPC-3 xenograft gave off brighter fluorescence than its neighborhood did. Furthermore, time-dependent accumulation of fluorescence to BxPC-3 tumor xenografts was observed and fluorescence in other parts was cleared gradually. At 72 hours after injection, significant fluorescence strength was detected mainly in the BxPC-3 tumor xenograft.  Figure 3(b) shows the* ex vivo* image of the dissected tumor xenograft of another mouse treated in the same way at 24 hours after injection. BxPC-3 xenograft showed rather strong fluorescence as compared with the PANC-1 xenograft.

### 3.4. Characterization of DOTA-Conjugated Antibodies

DOTA-conjugated 11-25 mAb or anti-KLH mAb was purified by column chromatography with PD-10. The average molecular weights of 11-25 mAb and DOTA-11-25 mAb determined by MALDI-TOF-MS were 151,599 Da and 153,038 Da, respectively. The average number of DOTA molecules conjugated per 11-25 mAb molecule was 3.7, as determined by dividing the mass difference between DOTA-11-25 mAb and 11-25 mAb by the molecular weight of DOTA (386 Da). In the same way, the average number of DOTA molecules per anti-KLH mAb was determined to be 4.8.

**Figure 4:**
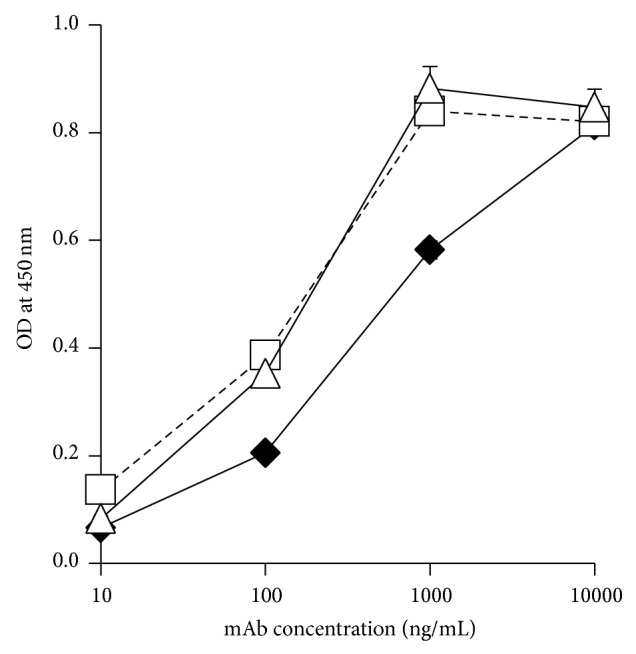
Binding activity of DOTA-conjugated 11-25 mAb and ^64^Cu-DOTA-11-25 mAb to recombinant MSLN determined by ELISA as compared with native 11-25 mAb. Native 11-25 mAb (open triangles) and DOTA-conjugated 11-25 mAb (open squares) and ^64^Cu-DOTA-11-25 mAb (closed diamonds) were added to the wells on which MSLN had been immobilized. Antibody binding was detected with peroxidase-labeled anti-mouse IgG and TMB. The reaction was stopped with 2 N H_2_SO_4_ and the absorbance at 450 nm was measured.

Binding of DOTA-conjugated 11-25 mAb to the MSLN protein showed that 11-25 mAb had a similar affinity to the MSLN after conjugation with DOTA (Figure 4).

### 3.5. *In Vitro* Characterization of ^64^Cu-DOTA-11-25 mAb

**Figure 5:**
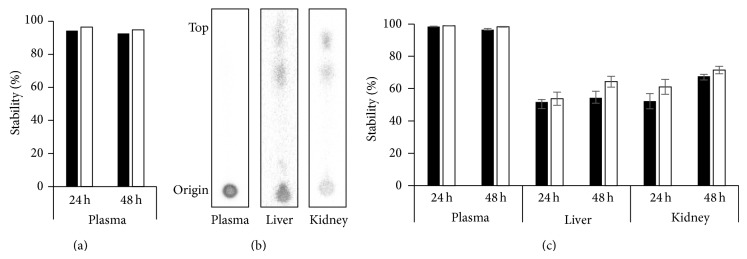
*In vitro* and* in vivo* stability of ^64^Cu-DOTA-11-25 mAb and ^64^DOTA-anti-KLH mAb. (a)* In vitro* stability of ^64^Cu-DOTA-11-25 mAb (black bars) and ^64^DOTA-anti-KLH mAb (white bars) after 24 hours and 48 hours of incubation at 37°C. Radioactivity derived from antibody-bound ^64^Cu stays at the origin and unbound ^64^Cu goes to the top of the chromatogram. The percentage of radioactivity that remained in the origin of TLC was shown as the stability. (b) Representative TLC-ARG of plasma and supernatant of homogenate from liver and kidney 24 hours after injection of ^64^Cu-DOTA-11-25 mAb. Aliquot of blood, liver, and kidney was taken from mice 24 hours after injection and analyzed by TLC-ARG as described in Section 2. (c)* In vivo* stability of ^64^Cu-DOTA-11-25 mAb (black bars) and ^64^Cu-DOTA-anti-KLH mAb (white bars) 24 and 48 hours after injection.

DOTA-conjugated 11-25 mAb and anti-KLH mAb were incubated with ^64^Cu and purified as described in Section 2. The radiochemical purity of resultant ^64^Cu-DOTA-mAbs determined by HPLC was 97.9% for 11-25 mAb and 100% for anti-KLH mAb. The specific activities of ^64^Cu-DOTA-11-25 mAb and ^64^Cu-DOTA-aKLH mAb were 0.74 and 0.82 MBq/*μ*g, respectively. The immunoreactivity retention after radiolabeling of 11-25 mAb against MSLN protein was measured by ELISA (Figure 4). At the concentration of 1000 ng/mL, the OD value of ^64^Cu-DOTA-11-25 mAb was 66.0% compared to that of native 11-25 mAb.* In vitro* stability in plasma after 24 and 48 hours of incubation analyzed by HPLC was 78.0% and 76.9% for ^64^Cu-DOTA-11-25 mAb and 82.8% and 77.9% for ^64^Cu-DOTA-anti-KLH mAb. The same preparations were also analyzed by TLC ARG (Figure 5(a)) and the results tended to be higher (94.3% and 92.6% for ^64^Cu-DOTA-11-25 mAb) than those analyzed by HPLC.

### 3.6. Cell Binding Assay with ^64^Cu-DOTA-11-25 mAb

**Figure 6:**
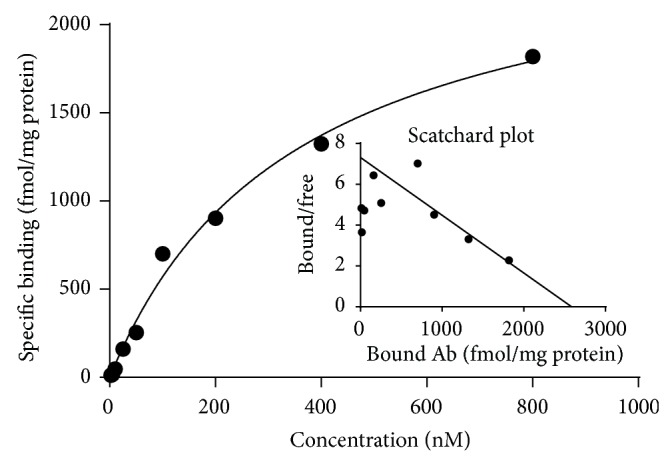
Cell binding assay with ^64^Cu-DOTA-11-25 mAb. BxPC-3 cells were transferred to 24-well plates at 5 × 10^4^ cells/well/mL and cultured for 4 days. Various concentrations of ^64^Cu-DOTA-11-25 mAb were incubated with the cell monolayers for 2 hours on ice in complete growth media (RPMI-1640 medium containing 10% fetal bovine serum). The cells were washed and lysed and their radioactivity was counted in a *γ*-counter. Inner graph showed the Scatchard plot of the specific binding versus the concentration of ^64^Cu-DOTA-11-25 mAb.

For* in vitro* experiment, ^64^Cu-DOTA-11-25 mAb was prepared and a binding study of ^64^Cu-DOTA-11-25 mAb with alive BxPC-3 cells was performed at 4°C. BxPC-3 cells exhibited saturable ^64^Cu-DOTA-11-25 mAb binding (Figure 6). The *K*_*D*_ of ^64^Cu-DOTA-11-25 mAb binding for the MSLN on BxPC-3 cells was 353 ± 63 nM.

### 3.7. PET Imaging and Biodistribution Studies

We performed PET imaging of ^64^Cu-DOTA-11-25 mAb in mice bearing BxPC-3, CFPAC-1, and PANC-1 tumors at 0, 24, and 48 hours after the intravenous injection. ^64^Cu-DOTA-anti-KLH mAb was used as a control. Figures 5(b) and 5(c) show the* in vivo* stability of ^64^Cu-DOTA-11-25 mAb and ^64^Cu-DOTA-anti-KLH mAb in the plasma, liver, and kidney at 24 hours and 48 hours after injection. Both of ^64^Cu-DOTA-11-25 mAb and ^64^Cu-DOTA-KLH mAb were stable in the blood until 48 hours after injection, and they were partly metabolized in liver and kidney extracts.

**Figure 7:**
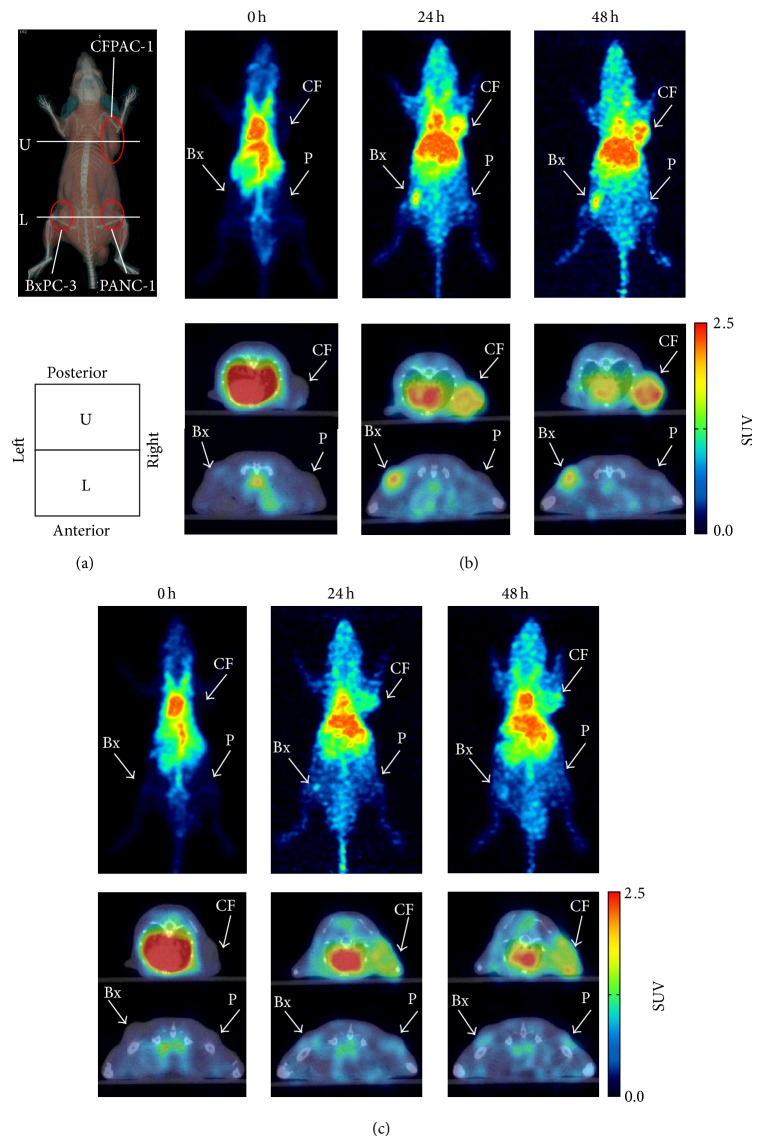
Representative PET images of mice bearing CFPAC-1, BxPC-3, and PANC-1. (a) A 3D volume rendering CT image of a mouse and a scheme showing the direction of PET images. The positions of xenografts and cross sections are shown on the CT image. CFPAC-1, BxPC-3, and PANC-1 xenografts were at the right shoulder, the left femur, and the right femur, respectively. (b) Serial PET/CT images of the nude mice at 0, 24, and 48 hours after administration of ^64^Cu-DOTA-11-25 mAb and (c) ^64^Cu-DOTA-anti-KLH mAb. Upper panels showed maximum intensity projections (MIP) of whole bodies. Lower panels are transverse PET/CT images at the position of tumors. Upper images (U) showed center of CFPAC-1 (CF), and lower images (L) showed BxPC-3 (Bx) and PANC-1 (P).

Figure 7 shows the representative result of the PET imaging.  Figure 7(a) is the 3D volume rendering image of CT of the mouse and shows the position of xenografts and the position of the cross section. In the upper cross section images of Figure 7(b), high accumulation of ^64^Cu-DOTA-11-25 mAb was observed in the CFPAC-1 xenograft (red arrowhead) at 24 and 48 hours. In the lower cross section images, ^64^Cu-DOTA-11-25 accumulation was observed in the BxPC-3 xenograft (yellow arrowhead) but not in the PANC-1 xenograft (white arrowhead). On the other hand, the accumulation of ^64^Cu-DOTA-anti-KLH mAb in CFPAC-1 and BxPC-3 xenografts (Figure 7(c)) was lower than those of ^64^Cu-DOTA-11-25 mAb.

**Figure 8:**
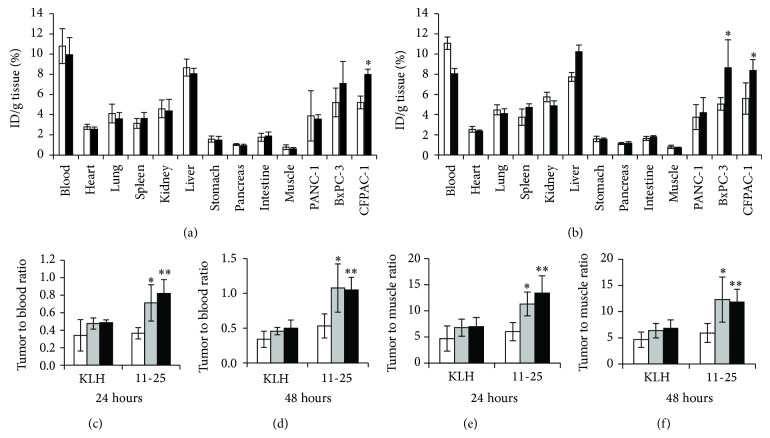
Biodistribution of ^64^Cu-DOTA-mAbs in mice bearing BxPC-3, CFPAC-1, and PANC-1 tumors at 24 hours (a) and 48 hours (b) after intravenous injection. The mice were sacrificed at 24 hours and 48 hours after intravenous injection of 11 MBq of ^64^Cu-DOTA-11-25 mAb (black bars) or ^64^Cu-DOTA-anti-KLH mAb (white bars). The organs were collected and weighed and radioactivity was measured by *γ*-counter. ^∗^ *P* < 0.05 versus the accumulation of ^64^Cu-DOTA-anti-KLH mAb. Tumor to blood ratio of ^64^Cu-lableld 11-25 mAb and ^64^Cu-DOTA-anti-KLH mAb in the tumors at 24 hours (c) and 48 hours (d) after injection of PANC-1 (white bars), BxPC-3 (gray bars), and CFPAC-1 (black bars). Tumor to muscle ratio of ^64^Cu-lableld 11-25 mAb and ^64^Cu-DOTA-anti-KLH mAb in the tumors at 24 hours (c) and 48 hours (d) after injection of PANC-1 (white bars), BxPC-3 (gray bars), and CFPAC-1 (black bars). The data were calculated as percentage of injected dose per gram of tissue (%ID/g). Mean and standard deviation have been corrected for physical decay of ^64^Cu. ^∗^ *P* < 0.05, ^∗∗^ *P* < 0.01 versus the ratio of PANC-1 tumor.

In the biodistribution study, relatively high accumulation of ^64^Cu-DOTA-11-25 mAb was observed in the blood, liver, and MSLN-positive tumors (Figure 8). In CFPAC-1 xenografts, the accumulation of ^64^Cu-DOTA-11-25 mAb was significantly higher than that of ^64^Cu-DOTA-anti-KLH mAb at both time points. In BxPC-3 xenografts, the accumulation of ^64^Cu-DOTA-11-25 mAb was significantly higher than that of ^64^Cu-DOTA-anti-KLH mAb at 48 hours after injection (Figures 8(a) and 8(b)). At both 24 and 48 hours after injection, the tumor to blood ratio and tumor to muscle ratio of ^64^Cu-DOTA-11-25 mAb in BxPC-3 tumor and in CFPAC-1 tumor were significantly higher than those in PANC-1 tumor (*P* < 0.05 and *P* < 0.01, resp., Figures 8(c)–8(f)). The average weight of individual PANC-1, BxPC-3, or CFPAC-1 tumor was 77 ± 49 mg, 50 ± 38/mg, or 207 ± 78 mg, respectively.* In vivo* stability of ^64^Cu-DOTA-11-25 mAb and ^64^Cu-DOTA-aKLH mAb was measured by TLC.

## 4. Discussion

The membrane-bound form of MSLN is present on a wide range of cancer cells [2–13] and its expression in normal tissues is relatively limited in the mesothelial cells. Soluble form of MSLN is found in the circulation in some cancer patients and monitoring the level of soluble MSLN is useful for diagnosis of malignant pleural mesothelioma [26, 32]. But the levels are too low (1.4–3.8 nmol/L) [26] to affect the* in vivo* targeting by anti-MSLN antibodies. With these properties of MSLN expression, MSLN can be a promising targeting molecule for a diverse range of cancers. Our aim is to develop a specific antibody-based PET probe that is available for detecting such a wide range of cancers. For that purpose, we selected 11-25 mAb as a candidate and confirmed that 11-25 mAb was available for detecting MSLN in Western blotting, flow cytometry, immunohistochemistry, and* in vivo* NIRF imaging.

Pancreatic cancer is one of the most common causes of cancer death. The high mortality rate of the pancreatic cancer is due to the high incidence of metastatic disease at initial diagnosis, the aggressive clinical course, and the lack of adequate systemic therapies. However, patients diagnosed at an early stage have the potential for therapeutic approach for prolonged survival. There is a need to develop a highly specific technique for early diagnosing. Accumulating evidence has shown that MSLN is overexpressed in various cancers, including pancreatic adenocarcinoma, ovarian cancer, and mesothelioma [3]. MSLN is an attractive candidate as a molecular target for pancreatic cancer marker-specific imaging or immunotherapy. So, we selected pancreatic adenocarcinoma cells, BxPC-3 and CFPAC-1, as an example set of target cells.

We first confirmed that 11-25 mAb can detect the soluble form of MSLN in the conditioned medium of pancreatic adenocarcinoma and mesothelioma (Table 1). Pancreatic adenocarcinoma cells, CFPAC-1 and BxPC-3, excreted soluble MSLN but epithelioid carcinoma, PANC-1, did not. Then we confirmed the expression of MSLN protein in these cells by Western blot and flow cytometry using 11-25 mAb and the expression of mRNA by semiquantitative reverse transcription PCR (Figure 1). The results were MSLN-positive for CFPAC-1 and BxPC-3 and negative for PANC-1, which were consistent with the soluble MSLN levels and previous reports [32, 33]. The immunohistochemistry results of these cell lines detected by 11-25 mAb showed that MSLN localized on the membrane and in the cytoplasm (Figure 2). On the culture slides or dishes, we observed that only a part of BxPC-3 cells, that is, the cells at the edge of cell clusters, was stained with 11-25 mAb. At the edge of the cluster the cells need to attach the new surface of dishes and proliferate. It was reported that MSLN plays a role in cell adhesion, migration, and proliferation [34–36]. This tendency is similar to the previous immunohistochemistry reports that the expression of MSLN was strongly observed in the invasive component of pancreatic cancer, but not in the noninvasive component even within the same specimen [4, 37, 38]. On the contrary, the xenografts of BxPC-3 and CFPAC-1 in mice were uniformly stained with 11-25 mAb (Figure 2(b)). Part of the reason is supposed to be that the human carcinoma cells were “invading” in mouse tissue and overexpressed MSLN.

As shown in Figure 3, we confirmed the specific accumulation of NIRF-conjugated 11-25 mAb in MSLN-positive BxPC-3 xenograft by* in vivo* fluorescent imaging. As the blood clearance of Alexa Fluor 750-labeled 11-25 mAb was slow, the contrast between MSLN-positive tumors and normal tissues was better at 48 hours after injection than at 24 hours after injection. NIRF has excellent optical characteristics in terms of stability, high sensitivity, and low autofluorescence. Although tissue penetration of NIRF is limited (up to several centimeters in tissues), NIRF-labeled antibody could be used for image-guided surgical resection of tumors in combination with preoperative PET imaging to detect the tumors [39].

For the detection of cancer, high-sensitivity imaging in deep tissue is necessary. PET imaging has the potential to meet the required sensitivity for tumors; besides it is noninvasive and is directed to the whole body. The radioactive metals are widely used for labeling peptides and antibodies by conjugation of metal chelators. ^64^Cu has a half-life of 12.7 hours and decays by 17.9% by *β*+ decay to ^64^Ni, 39.0% by *β*− decay to ^64^Zn, 43.1% by electron capture to ^64^Ni, and 0.475% *γ*-radiation/internal conversion. These emissions are 0.579 and 0.653 MeV for *β*− and positron, respectively, and 1.36 MeV for *γ*. ^64^Cu has been widely used for labeling antibodies for PET [40–42] and several clinical studies revealed that ^64^Cu-labeled IgG can detect tumors within 48 hours after injection [43, 44].

We used mono-N-hydroxysuccinimide ester 1, 4, 7, 10-tetraazacyclododecane-1, 4, 7, 10-tetraacetic acid (DOTA-mono-NHS ester) as a chelating agent for ^64^Cu. Although DOTA does not form very stable complex with ^64^Cu, several clinical studies revealed that ^64^Cu-labeled DOTA-IgG is adequately stable for PET imaging to detect lesions even around the liver [43]. Many structures, reported to form ^64^Cu-complexes with improved stability, such as TETA, TE2A, and CB-TE2A, require harsher reaction conditions to incorporate ^64^Cu. DOTA efficiently forms complexes with ^64^Cu under mild conditions adequate for radiolabeling biomolecules. In addition, although DOTA-conjugated therapeutic and diagnostic agents, such as ^90^Y-DOTA-TOC, are clinically approved, any other chelating agents which form ^64^Cu-complex are not.

The DOTA-11-25 mAb had similar reactivity to antigen MSLN, as compared with native 11-25 mAb. Further, ^64^Cu-DOTA-11-25 mAb kept the antigen specificity and bound to BxPC-3 cells* in vitro* with dissociation constant (*K*_*D*_) at 353 ± 63 nM (Figure 5).

^64^Cu-DOTA-11-25 mAb enabled visualization of MSLN-positive tumors by PET imaging. High accumulation of ^64^Cu-DOTA-11-25 mAb in MSLN-positive tumors was observed compared to that in the MSLN-negative tumor. As the tumor size of CFPAC-1 tumor was larger than the others, nonspecific accumulation of IgG probably by enhanced permeability and retention effect in the CFPAC-1 tumor was observed.

The biodistribution studies showed that the accumulation of ^64^Cu-DOTA-11-25 mAb in BxPC-3 and CFPAC-1 tumors was significantly higher than that in PANC-1 cells (Figure 8). However, the clearance of ^64^Cu-labeled IgG in the blood was very slow in general. Hassan et al. reported the biodistribution of ^111^In (*t*_1/2_ = 2.8 day)-K1 antibody in athymic nude mice bearing A-431-K5 (A-431 cells transfected with mesothelin gene) xenografts [31]. The peak uptake of ^111^In-K1 by A-431-K5 tumors was at 72 hours after injection. As the half-life of ^64^Cu is shorter than that of ^111^In, we only investigated the biodistribution at 24 and 48 hours after injection. Although the tumor to blood ratio was no more than 1, the ratio of ^64^Cu-DOTA-mAb in MSLN-positive tumors was significantly higher than that in MSLN-negative PANC-1 tumor. The accumulation of ^64^Cu-DOTA-11-25 mAb in the liver was relatively high, as it was previously reported for ^64^Cu-labeled IgG [42]. The presence of Fc receptor might have led to the nonspecific binding of IgG into the liver [45]. Transchelation of ^64^Cu to superoxide dismutase and metallothionein could also be a part of the reason for the accumulation in the liver [40]. As the distribution of ^64^Cu-DOTA-11-25 mAb in the pancreas or stomach was of very low level (Figure 7), MSLN-positive pancreatic cancer or gastric cancer could be visualized in high contrast by* in vivo* PET imaging with ^64^Cu-DOTA-11-25 mAb. Antibodies against human epidermal growth factor receptor 2 (HER2) have been approved for the treatment of HER2-positive breast cancer and they are used for companion diagnostic testing for targeted cancer therapies, as HER2-positive breast cancer is about 30% [46]. On the contrary, MSLN overexpression has been observed in a wide range of cancers; diagnostics with PET probe made from anti-MSLN antibody has the potential to be applicable to a broad spectrum of cancers.

Development of fragmented antibody derivatives such as Fab and single-chain Fv should be useful to achieve faster blood clearance and better “target-to-non-target” contrast at an early time point after administration relative to that of IgG 11-25 mAb.

In conclusion, 11-25 mAb specifically detected MSLN both in soluble form by ELISA and in cell-attached form by flow cytometry/immunohistochemistry and* in vivo* NIRF imaging. Further, we successfully labeled 11-25 mAb with ^64^Cu, with preservation of immunoreactivity. We evaluated the affinity of ^64^Cu-DOTA-11-25 mAb* in vitro* and used it as an* in vivo* PET imaging probe to detect MSLN-expressed pancreatic cancers. It was confirmed that ^64^Cu-labeled 11-25 mAb significantly accumulated in MSLN-expressing tumors compared to MSLN-negative tumor by PET imaging and biodistribution studies. As MSLN is highly expressed in various human tumors, our findings suggest that MSLN-specific imaging using ^64^Cu-labeled 11-25 mAb has the potential to be widely used in the diagnosis of patients with MSLN-positive cancers such as malignant mesotheliomas and ovarian cancers and pancreatic cancers.

## Figures and Tables

**Table 1:** Concentration of soluble MSLN in the culture media of cancer cells.

Tissue	Disease	Cancer cell line	Soluble MSLN (ng/mL)
Pancreas	Ductal adenocarcinoma	CFPAC-1	57.9
Pancreas	Adenocarcinoma	BxPC-3	72.6
Pancreas	Epithelioid carcinoma	PANC-1	0.0
Lung	Biphasic mesothelioma	MSTO-211H	71.6
Lung	Mesothelioma	NCI-H226	23.7
Lung	Squamous cell carcinoma	NCI-H520	0.0

The cells were cultured for 5 days and the culture media were collected and centrifuged. The supernatant was subjected to a sandwich ELISA for soluble MSLN, as described in Section 2.
